# Self-sacrifice in 'desperado' contests between relatives

**DOI:** 10.1186/1742-9994-7-27

**Published:** 2010-10-13

**Authors:** Adam L Cronin, Thibaud Monnin

**Affiliations:** 1 Laboratoire Écologie & Évolution CNRS UMR 7625 Université Pierre et Marie Curie 7 quai St Bernard, Bâtiment A 75 252 Paris, France

## Abstract

Intra-specific competition occurs in all animal species and can lead to escalated conflict. Overt fighting entails the risk of injury or death, and is usually avoided through the use of conventions or pre-fight assessments. However, overt fighting can be expected when value of the contest outweighs the value of the future, as contestants have little or nothing to lose. In these situations, respect for conventions and asymmetries between contestants can break down, and overt fighting becomes more likely (the desperado effect). Such conditions can arise in contests between queens over colony ownership in social insects, because the value of inheriting a colony of potentially thousands of helpers is huge and queens may have very limited alternative reproductive options. However, in social species the balance of possible outcomes may be influenced by inclusive fitness, as contestants are often relatives. Here we present a simple model based on social insects, which demonstrates that not fighting can be selectively advantageous when there is a risk posed by fighting to inclusive fitness, even when not fighting is likely to result in death. If contestants are related, a loser can still gain indirect fitness through the winner, whereas fighting introduces a risk that both queens will die and thereby obtain zero inclusive fitness. When relatedness is high and fighting poses a risk of all contestants dying, it can be advantageous to cede the contest and be killed, rather than risk everything by fighting.

## Background

Intra-specific contests, primarily over resources and reproductive opportunities, are a ubiquitous aspect of animal behaviour. Such contests are highly significant as they can determine a large proportion (or indeed all) of an animal's lifetime fitness, and pose the risk of injury or death when they escalate to fights. Because of these risks, contest resolution is often achieved without fighting, through processes of mutual and/or self-assessment [1-3]. However, models of animal contest predict that escalated contests leading to fatal fighting become more likely when the outcome of a contest represents a greater proportion of lifetime fitness [1,4]. That is, when contestants have little chance of fitness gains outside of the contest in question, fights should occur regardless of the risks involved because there is much to gain and little or nothing to lose [1,5]. Furthermore, kinship between contestants will not prevent escalated contests as long as the ratio between the value of the future and the value of the contest is small [4,5]. Fatal fighting can arise when *Vo*/*V *< 1 - *r*, where *Vo *and *V *are respectively the value of the future and of the current contest in terms of lifetime fitness, and *r *is the coefficient of relatedness between contestants [4]. Grafen [5] termed this the 'desperado effect', to describe the point where respect for conventions and asymmetries between contestants breaks down.

Escalated fighting occurs between relatives in a 'desperado' context in several circumstances: among male fig wasps and ants over mating opportunities [6-9], among nursery siblings over access to food resources in birds and mammals when young are overproduced [10,11], and among social insect queens over resource inheritance [3,12]. Contests are of utmost significance among queens of social insects as, in many species, colonies of potentially thousands of individuals are headed by one reproductive queen. Becoming queen thus represents a potentially huge fitness payoff, because once victorious, a queen is insulated from further environmental risks by the workers and can live for many years. Contests over the queen role can occur in normally monogynous species when several queens temporarily co-occur in one nest, such as during queen replacement, during colony reproduction via fission, and in pleometrotic foundress associations. In each case monogyny is soon restored: excess queens are culled by workers or killed during direct fighting between queens [12]. In such situations, there are usually very limited or no other reproductive opportunities for candidate queens. Thus, we would expect strong conflict between queens over colony inheritance to give rise to escalated fighting, and indeed this occurs in a range of species with variable degrees of relatedness between contestants (eg: honeybees [13,14], bumblebees [15], ants [16-18] and wasps [19]).

In the ant *Aphaenogaster senilis*, contests over colony inheritance occur during emergency queen replacement following orphaning. A few full-sister replacement queens are produced and the first-born queen is usually behaviourally dominant over later emerging queens [20]. Late-born queens apparently cede to the first-born queen without fighting and are eventually killed by workers. In experimental groups with two queens, second-born queens did not fight and usually died, even when the first-born had one mandible ablated and could not bite effectively [21]. Alternative reproductive opportunities for late-born queens are almost non-existent, and thus ceding to the first-born is effectively a form of suicide [21]. This lack of reciprocal fighting apparently contradicts the predictions of contest models.

However, models based on hawk-dove games generally assume that in an encounter between two hawks, one will survive while the other is killed. This does not take into account the possibility that both contestants die and thus obtain zero inclusive fitness. In do-or-die contests such as those over colony inheritance, it is possible that the victor sustains a mortal injury during the fight, and subsequently dies or is killed by workers, leading to the death of both contestants [eg: [21]]. Furthermore, as replacement queens are reared from existing queen-laid eggs in most social hymenoptera (workers typically cannot produce female brood), further queens cannot be reared once existing brood are exhausted, and there is a risk of colony death through failure to requeen.

A queen that cedes and is killed can still gain indirect fitness though the victorious queen if they are related, while at the same time avoiding the risk of a zero fitness outcome. Thus, the risk of both contestants dying may impose a significant cost to fighting if relatedness between the contestants is high [21]. Highly related contestants in 'desperado' contests, such as late-born *A. senilis *queens, may secure more indirect fitness by not fighting than direct fitness from victory when the latter is devalued by the risk of both queens dying [21]. Here, we formalise this reasoning by developing a simple model to explain how not fighting can be selectively advantageous in a 'desperado' contest between relatives. We focus on 'desperado' situations, where contestant's alternative options for fitness gain are negligible or absent. The area of contests outside of this has been explored by a suite of reproductive skew models (reviewed in [22]). Our model was developed to explain behaviour observed in social insect colonies, and we initially focus on these systems. The applicability of the rationale to other taxa is explored in the discussion.

### Life history of *A. senilis*

*A. senilis *is a monogynous, monandrous ant common to the Mediterranean basin, that reproduces by colony fission: queens are born with short wings and cannot fly, and only disperse with groups of workers on foot [23,24]. Colonies consist of around 1,300 workers and are monodomous. Colonies temporarily contain multiple queens when new queens are reared during preparation for colony fission or when the queen has died and must be replaced. Because of haplodiploidy, these new queens are full-sisters and are related by 0.75. The frequency of requeening in the field is unknown, but orphaning in the lab results in the production of one to six replacement queens: in one experiment 30 orphaned colonies produced 2.0 ± 1.1 queens [20], and in a second experiment 60 orphaned groups produced 2.8 ± 1.3 queens each [21]. New queens are produced 14-17 days apart on average. Only one queen survives, and it is usually the first-born who inherits the colony while subsequently born queens are killed [20,21].

Many strictly monogynous social insects go through transient polygynous phases: pleometrosis (founding of nests by multiple queens) occurs in many species as a regular mode of colony initiation [25], and fission occurs in a wide range of species, some of which are monogynous (eg: honeybees and army ants [26]). The frequency of queen replacement in nature is largely unknown, but it may be common in species that reproduce via fission (Cronin et al. unpublished data). Obligate monogyny in social insects is widespread, despite the fact that polygyny can be favoured for a variety of reasons including increased colony productivity, increased colony longevity, and high mortality of dispersing queens [[27], pp 126-134].

## Findings

### The model

We define our contest arena as a nest containing two queens and associated workers. This matches the experimental data on *A. senilis*, where the majority of contests during queen replacement are between two queens [20]. We assume monogyny and thus all but one queen will be killed. Reduction in queen number takes place via direct conflict between the queens if either queen initiates a contest, or by worker culling of one queen if neither queen initiates a fight. Queens cannot gain direct fitness by avoiding contest because queen selection will occur regardless of whether queens fight or not, and because queens have no reproductive opportunities outside the contest for colony ownership (thus *Vo *= 0; Table 1).

**Table 1:** Abbreviations and variables used in the model.

Variable	Range	Significance
Q1	na	The contestant holding the advantage in some form of asymmetry, which will thus win the contest if no overt fighting occurs.
Q2	na	The opponent, who will lose the contest unless it fights Q1.
*V*	na	Fitness value of the contest (ie: lifetime fitness of the queen that inherits the colony).
*Vo*	na	Fitness value of the future (ie: outside of the contest). *Vo *is here set to 0, as queens have no reproductive opportunities outside the contest.
*a*	0 to 1	The probability of Q1 winning an overt fight, here expressed as a linear function of relative fighting ability: *a *= ½(*f*+1)
*d*	0 to 1	The probability that fighting will result in the death of both queens, based on the mortality index and relative fighting ability, such that *d *=*m *for contestants of equal fighting ability, and declines with increasing disparity in fighting ability following *d *= *m.*e^-αf²^
*r*	0 to 1	Genetic relatedness between contestants.
*f*	-1 to 1	Relative fighting ability of Q1. When *f *= -1, Q1 has a lower fighting ability than Q2; when *f *= 0, Q1 and Q2 have equal fighting abilities; and when *f *= +1, Q1 has a higher fighting ability.
*m*	0 to 1	Mortality index. A factor based on the lethality of unrestrained combat for the species in question, expressed as a probability that a fight between contestants of equal fighting ability will lead to the death of both contestants.
α	≥0	A factor defining the shape of the curve describing the effect of relative fighting ability on the chance of mortality of both queens (Fig. 3).

For simplicity, we consider the contest in isolation, without regard to strategies employed in the rest of the population. We assume that the winning queen gains equal fitness (*V*) whether a fight occurs or not, as the duration of conflict (a few days) is negligible compared to the length of time fitness is gained (a few years), and non lethal injuries sustained during a fight have no effect on reproductive value since the queens accrue resources indirectly through workers. The effect of relaxing these assumptions is discussed later. We further assume that workers select queens based on some criteria of asymmetry between queens, as is common in species where intra-specific contests arise (eg: order of arrival or age [20,28,29]). This asymmetry gives one competitor some advantage, and the queen which holds the advantage in the asymmetry (Q1) will win the contest if no fighting occurs (workers will kill the other queen). The advantage may be uncorrelated with the individual's intrinsic quality [30], and we consider it independently of fighting ability and reproductive value. In *A. senilis*, the asymmetry is determined by birth order, and the first born queen (Q1) generally inherits. How workers select one queen over the other is generally unclear, but may be related to a need to expedite the queen replacement process [20,21], or because of queen manipulation [31,32].

The possible contest outcomes are thus a) Q1 inherits the colony, either through killing Q2, or when Q2 is killed by workers in the case that no queen-queen fight occurs, b) Q2 inherits the colony by killing Q1, or c) both queens are killed fighting. Queens can gain direct fitness through inheriting the colony or indirect fitness when a relative inherits the colony. The fitness payoff for a given queen depends on the probability of winning the fight if they do fight, the risk of both queens dying, and the genetic relatedness between the queens.

Both queens may either choose to fight or refrain from fighting, and we assume a fight occurs if one queen initiates fighting. Q1 obtains a direct fitness of *V *if there is no fighting (workers kill Q2; see Table 1 for definitions of variables used in the model). When fighting occurs, Q1 has an inclusive fitness of:

(1) (aV+(1–a)r V) (1–d)

Where *a *is the probability of Q1 winning an overt fight and 1 - *a *is the probability of Q2 winning. Genetic relatedness between contestants is denoted by *r*, and *d *represents the probability of a zero fitness outcome because both queens are killed in the contest.

From the above, it can be seen that Q1 prefers not to fight when:

(2) V>(a V+(1–a)r V)(1–d)

Q2 obtains no direct fitness if she elects not to fight (workers kill her), but she obtains an indirect fitness of *r V*. If Q2 fights, her inclusive fitness is (*a r V *+ (1 - *a*) *V*) (1 - *d*). Thus, Q2 favours not fighting when:

(3) rV>(a r V+(1–a)V)(1–d)

Solving these inequalities yields the values for which not fighting is favoured by Q1 {2} and Q2 {3}. From {2} it is clear that Q1 never prefers fighting, which is intuitive as she stands to inherit if there is no fighting. For Q2, the decision to fight or not is independent of the value of the contest (*V*) but dependent on the probability of winning (*a*), relatedness (*r*) and the probability of a zero fitness outcome (*d*). Q2 prefers not to fight when:

(4) d>(1–r)(1–a)/(1–a+ar)

**Figure 1:**
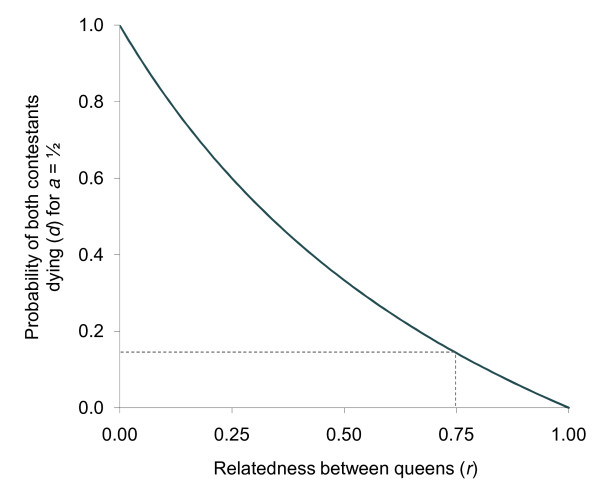
**Conditions favouring not fighting by Q2 for contestants of equal fighting ability**. Critical values of *d *for which not fighting (above the line) or fighting (below the line) is favoured, for all values of *r*, from equation {4}. When fighting abilities are equal (*a *= ½), the decision whether to fight or not is a function of *d *and *r*. This figure indicates that Q2 increasingly favours not fighting for increasing values of *r *and *d*. When contestants are full sisters (*r *= 0.75), Q2 will not fight unless the risk of both queens dying is very low (*d *< 0.143).

When queens are close relatives, have developed in the same environment, and have been fed equally, they may have an equal probabilities of winning a fight (i.e. *a *= 0.5), in which case {4} becomes *d *>(1 - *r*)/(1 + *r*). This relationship is depicted in Fig. 1 and indicates that when queens are full sisters (*r *= 0.75), as in *A. senilis*, Q2 fights only if the probability of both queens dying is low (*d *< 1/7 (= 0.143)).

**Figure 2:**
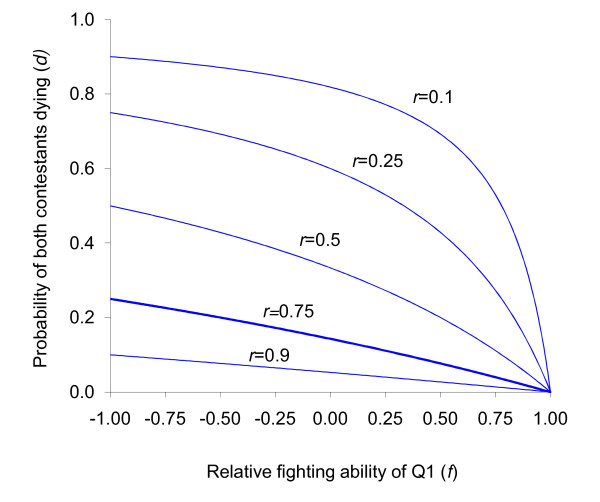
**Conditions favouring not fighting by Q2**. Critical values of *d *for which not fighting (above the lines) or fighting (below the lines) is favoured by Q2 for various values of *f*, from equation {4}. Each line represents a value of relatedness (*r *= 0.1, 0.25, 0.5, 0.75 and 0.9, with *r *= 0.75 (full sisters) indicated by a thick line). For clone queens (*r *= 1), critical values are always 0, and not fighting is always favoured. Not fighting is favoured when relatedness is high or when Q1 is a better fighter.

However, fighting asymmetries between contestants are likely if size differences exist or if differences in eclosion time results in development related fighting advantages. We explore this possibility by defining the probability of Q1 winning an overt fight as a linear function of Q1's relative fighting ability *f*, with *a*(*f*) = ½ (*f *+ 1). Equation {4} then becomes: *d *>(1 - *f*)/[1 - *f *+ 2*r*/(1 - *r*)] (Fig. 2). In this scenario, Q2 prefers not to fight when relatedness is high, and fighting asymmetry has little impact on this preference. However, when relatedness is low the effect of the fighting asymmetry is markedly stronger when it is in favour of Q1: being the better fighter only marginally increases Q2's propensity to fight whereas being the poorer fighter can dramatically reduce it (for low values of *r*). As it is likely that any asymmetry is in favour of the first-born [eg: [21,33]], increasing fighting asymmetry can have the effect of reducing Q2's propensity to fight.

**Figure 3:**
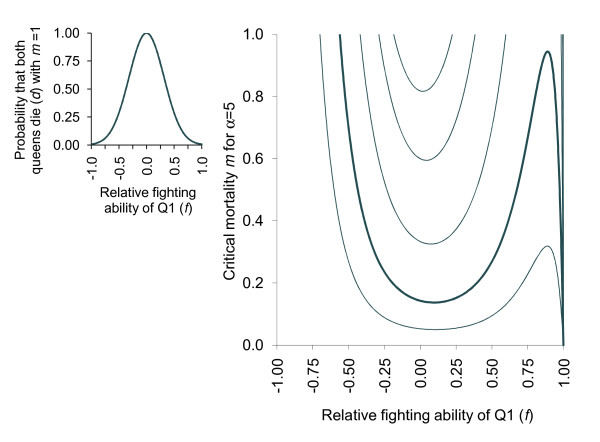
**Mortality risk curve for α = 5, and corresponding conditions favouring not fighting by Q2**. Effect of relative fighting ability of Q1 (*f*) on diminishing the probability that overt fighting leads to the death of both contestants following *d *= *m *e^-αf²^. The inset graph shows the distribution of the probability of both queens dying for *m *= 1 and α = 5. The risk of mortality of both queens is restricted to a smaller range of *f *values, and is close to zero for extremely biased fighting ability. The main figure shows that when the risk to inclusive fitness decreases with increasing asymmetry in fighting ability, Q2 is more likely to fight, particularly when it is a relatively strong fighter. Not fighting remains the preferred strategy when fighting represents a risk to inclusive fitness.

Finally, it is worth considering that relative fighting ability affects not only the probability of winning the fight, but also the risk that both queens die: more evenly matched queens are more likely to have longer fights leading to a higher risk of both queens dying. In this case, the risk of both queens dying *d *becomes a diminishing function of increasing relative fighting ability. We can define *d *as a function of Q1's relative fighting ability *f *and a mortality index *m*, following *d*(*f*) = *m *e^-αf²^. The variable *m *describes the lethality of fights in a given species while α defines the shape of the distribution (Table 1). Inequality {4} then becomes *m *>(1 - *f*)/[(1 - *f *+ 2*r*/(1 - *r*))e^-αf²^]. Fig. 3 shows *d*(*f*) for α = 5, and the critical mortality above which Q2 does not fight. When queens have similar fighting abilities Q2 still prefers not to fight, but increasing disparity in fighting ability now increases Q2's propensity to fight, because the risk of a zero fitness outcome is reduced. Overall, Figs. 1 and 3 show that Q2 favours not fighting when there is a risk to her inclusive fitness: when the risk that both queens die from fighting is restricted to when queens have similar fighting abilities, the preference for not fighting by Q2 becomes similarly restricted. Thus, while the threat of a zero fitness outcome can favour not fighting by subordinate queens, large differences in fighting ability can negate this.

## Discussion

Overt fighting can be expected in animal contests when the value of the contest is high relative to the value of the future [4] as contestants have nothing to lose. However, in contests between relatives there *is *something to lose, because fighting poses the risk of both contestants dying: in fighting with a related opponent, a losing individual can risk inclusive fitness loss by fatally injuring its competitor. Our results suggest that relatedness between contestants can lead to restraint under specific circumstances, even when not fighting means death and there are no alternative future reproductive opportunities. Our model indicates that this is possible when three conditions are met: i) contests are 'desperado' contests, with one winner and no alternative opportunities for fitness gains for the loser, ii) fighting poses a risk of mortality to the victor and, iii) contestants are highly related.

We have assumed that Q2 decides for herself how to act in a given situation. An alternative possibility is that her behaviour is manipulated by Q1 and/or workers, both of which benefit from Q1 inheriting if she is fit. It is unclear whether Q1 has the means of manipulating Q2 (eg: through chemical signals), but workers presumably do, as they control the amount of food Q2 receives as a larva, which may allow them to control her growth rate and hence delay her emergence, thereby advantaging Q1. Mechanisms of influencing offspring development and contest outcomes have also been described in vertebrates [33]. However, whether Q2 is manipulated or not in her decision to fight in *A. senilis *awaits investigation.

We employ the simplifying assumption of considering our contest in isolation, without regard to population-wide strategies. In reality, model parameters such as the value of the contest *V *are not independent of strategies employed by other colonies in the population, and this should be considered in a more comprehensive model. Furthermore, we assume a 2-queen contest arena and, while our model is applicable to most *A. senilis *colonies where only a few queens are produced, in some colonies Q1 may need to win multiple consecutive contests, and this may be the norm in other species. In such cases, an injury sustained in a fight will lower the relative fighting ability of the queen for the next fight. For instance, if Q1 wins the fight but is injured in the process she may have a lower fighting ability against a third queen. Nonetheless, unless Q3 is a markedly better fighter, she will still elect not to fight in order not to jeopardize her inclusive fitness (Fig. 3 with -0.25 <*f *<0). Conversely, if Q3 is a much stronger fighter (*f *< - 0.4) there is little risk that the queens will kill one another and Q3 will fight. However, in *A. senilis *late born queens do not fight even when Q1 has had one mandible ablated and cannot bite effectively (ie: *f *is close to -1) [21]. It may be that in this species, queens have not been selected to assess fighting abilities because queens are full-sisters and differ too little in fighting ability to ever make fighting a successful strategy for Q2 (Fig. 1).

Clearly, the conditions required for self sacrifice to occur are restrictive, and probably do not arise in most cases of animal contests because i) relatedness between contestants is insufficiently high or ii) there are other opportunities for fitness acquisition outside the focal contest. This includes contests among ant queens following pleometrosis: as foundresses are usually unrelated [25] overt fighting eventuates (eg: *Lasius niger *[16,18], *Messor pergandei *[34], *Azteca xanthacroa *[35], *Solenopsis wagneri *(monogyne form) [12,17]).

Conditions potentially giving rise to desperado contests between relatives can arise in several contexts in insects: among queens fighting over colony inheritance in monogynous species that can requeen or reproduce by colony fission (eg: *A. senilis *[20,21] and honeybees [3], see also Table 2), and among wingless males of *Cardiocondyla *and *Hypoponera *ants, which may fight until only one remains in the colony and monopolises mating opportunities [6,7,36,37]. However, the available evidence suggests overt fighting is usually the norm, possibly because relatedness between contestants is low as a result of polygyny and polyandry (Table 2).

**Table 2:** Examples of contests between relatives in a potential 'desperado' context in social insects.

	Species	Contestants	Reason for contest	Relatedness to competitors	Overt reciprocal fighting?	References
	*Aphaenogaster senilis*	Queens	Colony inheritance during requeening	High (0.75)	N	[20,21]
	*Cataglyphis cursor*	Queens	Colony inheritance during requeening or fission	Low (polyandry) to high (parthenogenesis)	Y	[43], Chéron unpublished data
Ants	*Cardiocondyla wroughtonii*	Males	Mating opportunities	Low (polygyny)	Y	[6,7]
	*Hypoponera bondroiti*	Males	Mating opportunities	Low (polygyny)	Y	[44]

	*Apis mellifera*	Queens	Colony inheritance during requeening or fission	Low (polyandry)	Y	[13,14]
Bees	*Melipona beecheii*	Queens	Normal colony cycle	Moderate (mother - daughter) to High (full-sisters)	N (workers kill new queens quickly)	[42,45]
	*Pleibia remota*	Queens	Colony inheritance during colony cycle	Moderate?	Y (mother and daughter)	[46]

In most instances relatedness is lower than in *A. senilis*, which may explain the prevalence of fighting reported in other species. The transitory presence of multiple related queens arises in monogynous social insects during preparation for colony fission, queen replacement, and as part of the normal colony cycle in stingless bees (continuous rearing). Male-male contests arise when mating occurs in the natal nest.

Males also fight over females in many fig wasps [8,9,38], where the intensity of fights varies between species from causing no injury [9] to causing a high level of life-threatening injuries [38,39]. Here, self-sacrifice is not expected because competing males are not highly related in many species [38] and males may avoid fights they are unlikely to win [39].

In vertebrates, siblings fight over food in some birds and mammals producing more young than can be reared [40]. However, in many cases these may not be true desperado contests as all young may be reared in exceptionally bountiful years, even in 'obligately' siblicidal species [11,41]. Nonetheless, examples of overt fighting leading to siblicide exist for a range of avian species and some mammals [[41] and references therein], in which junior young are usually killed by senior siblings. Conditions favouring self-sacrifice may not arise because relatedness between vertebrate siblings (*r *= 0.5) is lower than for Hymenopteran full-sisters (*r *= 0.75) and, furthermore, development related asymmetries may be so extreme that the risk of contestants mortally injuring one another is negligible (ie: there is no risk to inclusive fitness), rendering self-sacrifice irrelevant. We predict that circumstances that can give rise to self-sacrifice are most likely to be found in monogynous and monandrous species of social insect that can requeen or that reproduce by colony fission, as relatedness is high and death of all but one contestant is inevitable. Stingless bees (Meliponini) fulfil these criteria, but queen-queen contests can be precluded by worker pre-emptively culling of queens [42] and details of queen-queen conflict are presently lacking. Closer investigation of contests in species where the specific conditions described arise could demonstrate further examples of self-sacrifice.

## Competing interests

The authors declare that they have no competing interests.

## Authors' contributions

Both authors contributed to the manuscript and the development of the model, and approved the final manuscript.

## References

[B1] Maynard-Smith JM Price GR Logic of Animal Conflict Nature 1973 246 15 18 10.1038/246015a0

[B2] Parker GA Assessment strategy and evolution of fighting behavior J Theor Biol 1974 47 223 243 10.1016/0022-5193(74)90111-8 4477626

[B3] Dietemann V Zheng HQ Hepburn C Hepburn HR Jin SH Crewe RM Radloff SE Hu FL Pirk CWW Self assessment in insects: honeybee queens know their own strength PLoS ONE 2008 3 e1412 10.1371/journal.pone.0001412 18183293 PMC2173938

[B4] Enquist M Leimar O The evolution of fatal fighting Anim Behav 1990 39 1 9 10.1016/S0003-3472(05)80721-3

[B5] Grafen A The logic of divisively asymmetric contests - respect for ownership and the desperado effect Anim Behav 1987 35 462 467 10.1016/S0003-3472(87)80271-3

[B6] Kinomura K Yamauchi K Fighting and mating behaviors of dimorphic males in the ant *Cardiocondyla wroughtoni* J Ethol 1987 5 75 81 10.1007/BF02347897

[B7] Stuart RJ Francoeur A Loiselle R Lethal fighting among dimorphic males of the ant, *Cardiocondyla wroughtonii* Naturwissenschaften 1987 74 548 549 10.1007/BF00367076

[B8] Hamilton WD Blum MS, Blum NA Wingless and fighting males in fig wasps and other insects Sexual Selection and Reproductive Competition in Insects 1979 New York: Academic Press 167 220

[B9] Greeff JM van Noort S Rasplus JY Kjellberg F Dispersal and fighting in male pollinating fig wasps CR Biol 2003 326 121 130 10.1016/S1631-0691(03)00010-6 12741187

[B10] Hofer H East ML Siblicide in Serengeti spotted hyenas: a long-term study of maternal input and cub survival Behav Ecol Sociobiol 2008 62 341 351 10.1007/s00265-007-0421-3

[B11] Drummond H Rodriguez C Vallarino A Valderrabano C Rogel G Tobon E Desperado siblings: uncontrollably aggressive junior chicks Behav Ecol Sociobiol 2003 53 287 296

[B12] Balas MT Conditions favoring queen execution in young social insect colonies Insect Soc 2005 52 77 83 10.1007/s00040-004-0774-9

[B13] Gilley DC The behavior of honey bees (*Apis mellifera **ligustica*) during queen duels Ethology 2001 107 601 622 10.1046/j.1439-0310.2001.00692.x

[B14] Tarpy DR Mayer MK The effects of size and reproductive quality on the outcomes of duels between honey bee queens (Apis mellifera L.) Ethol Ecol Evol 2009 21 147 153 10.1080/08927014.2009.9522503

[B15] Cameron SA Jost MC Mediators of dominance and reproductive success among queens in the cyclically polygynous Neotropical bumble bee *Bombus atratus *Franklin Insect Soc 1998 45 135 149 10.1007/s000400050075

[B16] Sommer K Hölldobler B Colony founding by queen association and determinants of reduction in queen number in the ant *Lasius niger* Anim Behav 1995 50 287 294 10.1006/anbe.1995.0244

[B17] Bernasconi G Keller L Reproductive conflicts in cooperative associations of fire ant queens (*Solenopsis invicta*) Proc R Soc Lond B 1996 263 509 513 10.1098/rspb.1996.0077

[B18] Aron S Steinhauer N Fournier D Influence of queen phenotype, investment and maternity apportionment on the outcome of fights in cooperative foundations of the ant *Lasius niger* Anim Behav 2009 77 1067 10.1016/j.anbehav.2009.01.009

[B19] Nascimento FS Tannure-Nascimento IC Zucchi R Behavioral mediators of cyclical oligogyny in the Amazonian swarm founding wasp *Asteloeca ujhelyii *(Vespidae, Polistinae, Epiponini) Insect Soc 2004 51 17 23 10.1007/s00040-003-0696-y

[B20] Chéron B Doums C Fédérici P Monnin T Queen replacement in the monogynous ant *Aphaenogaster senilis*: supernumerary queens as life insurance Anim Behav 2009 78 1317 1325 10.1016/j.anbehav.2009.08.016

[B21] Cronin AL Monnin T Bourgeois queens and high stakes games in the ant *Aphaenogaster senilis* Front Zool 2009 6 24 10.1186/1742-9994-6-24 19840383 PMC2771002

[B22] Buston PM Zink AG Reproductive skew and the evolution of conflict resolution: a synthesis of transactional and tug-of-war models Behav Ecol 2009 20 672 684 10.1093/beheco/arp050

[B23] Ledoux A Un nouveau mode de bouturage de société chez la fourmi *Aphaenogaster senilis *Mayr CR Acad Sci Ser D 1971 273 83 85

[B24] Boulay R Hefetz A Cerdá X Devers S Francke W Twele R Lenoir A Production of sexuals in a fission-performing ant: dual effects of queen pheromones and colony size Behav Ecol Sociobiol 2007 61 1531 1541 10.1007/s00265-007-0385-3

[B25] Bernasconi G Strassmann JE Cooperation among unrelated individuals: the ant foundress case Trends Ecol Evol 1999 14 477 482 10.1016/S0169-5347(99)01722-X 10542454

[B26] Peeters C Ito F Colony dispersal and the evolution of queen morphology in social Hymenoptera Annu Rev Entomol 2001 48 601 630 10.1146/annurev.ento.46.1.601 11112181

[B27] Crozier RH Pamilo P Evolution of Social Insect Colonies 1996 Oxford: Oxford University Press

[B28] Seppä P Queller DC Strassmann JE Reproduction in foundress associations of the social wasp, *Polistes carolina*: conventions, competition, and skew Behav Ecol 2002 13 531 542 10.1093/beheco/13.4.531

[B29] Bridge C Field J Queuing for dominance: gerontocracy and queue-jumping in the hover wasp *Liostenogaster flavolineata* Behav Ecol Sociobiol 2007 61 1253 1259 10.1007/s00265-007-0355-9

[B30] Maynard Smith J Evolution and the Theory of Games 1982 Cambridge, UK: Cambridge University Press

[B31] Monnin T Ratnieks FLW Jones GR Beard R Pretender punishment induced by chemical signalling in a queenless ant Nature 2002 419 61 65 10.1038/nature00932 12214231

[B32] Tarpy DR Fletcher DJC "Spraying" behavior during queen competition in honey bees J Insect Behav 2003 16 425 437 10.1023/A:1024884211098

[B33] Drummond H Rodriguez C Schwabl H Do mothers regulate facultative and obligate siblicide by differentially provisioning eggs with hormones? Journal of Avian Biology 2008 39 139 143 10.1111/j.0908-8857.2008.04365.x

[B34] Rissing SW Pollock GB Social-interaction among pleometrotic queens of *Veromessor pergandei *(Hymenoptera, Formicidae) during colony foundation Anim Behav 1986 34 226 233 10.1016/0003-3472(86)90027-8

[B35] Choe JC Perlman DL Choe JC, Crespi BJ Social conflict and cooperation among founding queens in ants (Hymenoptera: Formicidae) The Evolution of Social Behavior in Insects and Arachnids 1997 Cambridge: Cambridge University Press 392 406 full_text

[B36] Heinze J Hölldobler B Yamauchi K Male competition in *Cardiocondyla *ants Behav Ecol Sociobiol 1998 42 239 246 10.1007/s002650050435

[B37] Yamauchi K Ishida Y Hashim R Heinze J Queen-queen competition by precocious male production in multiqueen ant colonies Curr Biol 2006 16 2424 2427 10.1016/j.cub.2006.10.007 17174916

[B38] West SA Murray MG Machado CA Griffin AS Herre EA Testing Hamilton's rule with competition between relatives Nature 2001 409 510 513 10.1038/35054057 11206546

[B39] Moore JC Obbard DJ Reuter C West SA Cook JM Fighting strategies in two species of fig wasp Anim Behav 2008 76 315 322 10.1016/j.anbehav.2008.01.018

[B40] Mock DW Parker GA The Evolution of Sibling Rivalry 1997 Oxford: Oxford University Press

[B41] Osorno JL Drummond H Is obligate siblicidal aggression food sensitive? Behav Ecol Sociobiol 2003 54 547 554 10.1007/s00265-003-0667-3

[B42] Wenseleers T Hart AG Ratnieks FLW Quezada-Euan JJG Queen execution and caste conflict in the stingless bee *Melipona beecheii* Ethology 2004 110 725 736 10.1111/j.1439-0310.2004.01008.x

[B43] Pearcy M Aron S Doums C Keller L Conditional use of sex and parthenogenesis for worker and queen production in ants Science 2004 306 1780 1783 10.1126/science.1105453 15576621

[B44] Yamauchi K Kimura Y Corbara B Kinomura K Tsuji K Dimorphic ergatoid males and their reproductive behavior in the ponerine ant *Hypoponera bondroiti* Insect Soc 1996 43 119 130 10.1007/BF01242564

[B45] Paxton RJ Weißschuh N Engels W Hartfelder K Quezada-Euan JJG Not only single mating in stingless bees Naturwissenschaften 1999 86 143 146 10.1007/s001140050588

[B46] Imperatriz-Fonseca VL Zucchi R Virgin queens in stingless bee (Apidae, Meliponinae) colonies: a review Apidologie 1995 26 231 244 10.1051/apido:19950305

